# Recent developments and strategies in pediatric pharmacology research in the USA

**DOI:** 10.1186/1753-2000-2-36

**Published:** 2008-12-08

**Authors:** Benedetto Vitiello

**Affiliations:** 1 Child and Adolescent Treatment and Preventive Intervention Research Branch, Division of Services and Intervention Research, National Institute of Mental Health, Bethesda, Maryland, USA

## Abstract

Research in pediatric pharmacology has undergone major changes in the last ten years, with an expansion in both publicly and privately funded activities. A number of pharmacokinetics studies and multi-site controlled efficacy trials have been conducted, so that treatment of children and adolescents can now be better informed and evidence-based. Regulatory financial incentives to industry in return for studies on drugs still covered by patent exclusivity have resulted in a substantial increase in pediatric research funded by pharmaceutical companies. In parallel, public funding has supported research on off-patent medications and other clinical important aspects of treatment, such as comparisons between active treatments, including non-pharmacological interventions. With greater interest by industry in pediatric research, the role of government funding agencies has been redefined to avoid duplication and ensure better integration of efforts and utilization of resources. The present review discusses some of the recent developments in pediatric pharmacology with focus on psychiatric medications.

## Background

The last ten years have witnessed both a significant expansion of pediatric pharmacology research and a redefinition of the roles of public and private sources in supporting such research. Traditionally a neglected area of medicine, research on medication effects in children was brought to the center of the attention in the 1990s [1,2]. The realization that the pediatric use of medications was expanding without adequate evidence for efficacy and safety spurred a number of initiatives in the U.S. [3,4].

On one hand, the National Institutes of Health (NIH) established research networks devoted to testing in children the pharmacokinetics, efficacy, and safety of commonly prescribed medications that were approved by the U.S. Food and Drug Administration (FDA) only for adult use ("off-label use") [5,6]. On the other hand, legislation was enacted to provide incentives to the pharmaceutical industry for sponsoring pediatric studies [7-10]. As a consequence, a variety of funding sources and possible strategies has become available for conducting systematic investigations in child and adolescent pharmacology. The aim of this review is to discuss some of the recent developments in pediatric pharmacology research in the U.S.A., and examine strategies and approaches to conducting investigations in children with special focus on psychopharmacology.

### Publicly funded research

Most of the funding in pediatric pharmacology research by the NIH is provided through investigator-initiated grant applications, which undergo rigorous peer-review. Over the years, this mechanism has supported a large part of pharmacology research relevant to children. In fact, prior to the legislative changes of 1997 [7], industry had little interest in pediatric research, even in the case of widely used medications. For example, the first trial showing the efficacy of fluoxetine in childhood depression was funded through a public grant [11].

Besides funding investigator-initiated research, NIH launched a number of initiatives in areas of high public health significance. In the mid-1990s, research networks were started to specifically investigate pediatric pharmacology. The Pediatric Pharmacology Research Units (PPRUs) and the Research Units on Pediatric Psychopharmacology (RUPPs) were funded by the National Institute of Child Health and Human Development (NICHD) and the National Institute of Mental Health (NIMH), respectively [4,5]. The PPRUs have focused on evaluating the pharmacokinetics and efficacy of drugs commonly used in general pediatrics, and thus provided an infrastructure of highly specialized academic research settings where both NIH- and industry-funded studies can be conducted [12,13].

Also based at academic sites, the RUPPs conducted controlled clinical trials of the efficacy of psychiatric medications used off-label in the community, such as the selective serotonin reuptake inhibitor (SSRI) fluvoxamine for the treatment of pediatric anxiety disorders and the antipsychotic risperidone in children with autism and severe behavioral problems [14,15]. In addition to research networks, NIMH has funded multisite clinical trials to address specific questions of high clinical relevance to the treatment of children with attention deficit/hyperactivity disorder (ADHD), adolescent depression, early onset schizophrenia, anxiety disorders, and bipolar disorder [5,16-20].

The increasing interest of industry in pediatric research that had been traditionally supported by NIH had led to a redefinition of the role of public funding, which is now focused on investigating important aspects of pharmacology that go beyond traditional pharmacokinetics or efficacy testing versus placebo. In particular, comparisons of the effectiveness of alternative specific interventions and treatment strategies have been the object of recent NIMH-funded clinical trials with the aim of providing clinicians and families with the data necessary for informed treatment decisions. Thus, in the case of ADHD and adolescent depression, conditions for which both pharmacological and psychotherapeutic interventions are available, NIMH funded large trials to compare the effectiveness of these treatments used as monotherapy or in combination [16,21]. Likewise, a publicly funded comparative trial of different antipsychotics for youths with schizophrenia was recently completed [20]. Some of these studies have also produced informative cost-effectiveness analyses that should help better allocate health care resources [22,23].

Evaluating the effectiveness of interventions in clinical settings requires an investigational approach and a research infrastructure that are different from those of the traditional efficacy trials, which are typically conducted in research settings and are characterized by high internal but low external validity [24]. To this end, efforts are under way to create a child psychiatry practice-based network where observational studies and pragmatic clinical trials could be conducted in a rapid and efficient way [25,26].

Other areas relevant to pediatric pharmacology that have received little attention from industry are: a) the development of treatments for autism, other pervasive developmental disorders, and rare disorders such as Tourette's disorder and child schizophrenia; b) pharmacoepidemiology research; c) meta-analyses; d) bioethics research; e) development of novel methodological tools to assess treatment effects and safety; and f) evaluations of the impact of treatment on long-term illness trajectories and distal prognosis [4,27]. It is evident that the research agenda in pediatric pharmacology now extends well beyond the traditional and essential component of pharmacokinetics and efficacy testing of efficacy, and involves, among the others, comparative studies of treatment strategies, systematic analyses of community patient databases, and active post-marketing surveillance for a more effective pharmacovigilance. Finally, as discussed more in details further down, a specific role of NIH under the Best Pharmaceuticals for Children Act of 2002 [8] is to support pediatric studies of off-patent drugs, thus integrating and complementing the exclusivity extension initiative that applies to patented drugs.

### Industry-funded research

As mentioned, the involvement of industry in pediatric research had been rather limited until the late 1990s. This situation substantially changed in the U.S. with the enactment of legislation providing pharmaceutical companies with an additional six months of drug patent exclusivity protection in return for conducting specific studies in children [7]. The legislation, initially enacted in 1997, and then confirmed and expanded in 2002 and 2007 [8,10], has provided a powerful incentive for industry-funded pediatric research.

For example, industry has funded most of the placebo-controlled efficacy trials of stimulants and antidepressants during the last ten years, thus providing the data for recent meta-analyses conducted by the FDA and academic researchers [28-30].

The exclusivity extension program has been successful in stimulating industry-funded research in pediatric pharmacology in the U.S. [31]. Knowledge of the medication dosing appropriate to children has been expanded [32]. From 1998 through May 2008, more than three hundred studies have been conducted and 148 changes in drug labeling implemented consequent to research conducted by industry under the pediatric exclusivity program [33]. About half of these studies were aimed at testing efficacy, a third at examining pharmacokinetics, and one fifth at evaluating safety.

The overall positive result of this legislative is reflected in the decision of the European Union to enact similar incentives to industry for pediatric research [34,35].

Independent analyses of the economic cost and return to industry of the pediatric exclusivity program have been reported [36]. When research conducted on nine drugs was considered, the cost to industry ranged from $5 to $44 million, with a median of $12.3 million. The net return ranged from $9 to $508 million, with a median of $140 million, and the ratio return/cost went from -0.68 to 73.63. Obviously, the return depends on the magnitude of the overall market of the drug, including both pediatric and adult market. Thus, in the case of a widely prescribed antidepressant, pediatric studies with a cost of $35 million translated into a return of $242 million, for return/cost ratio of 7 [36].

Notwithstanding the overall success of the pediatric exclusivity program in fostering research, a number of limitations have been identified and several concerns raised, some of which have been addressed during the re-authorization of the program. Because the exclusivity is granted for completing specific studies within a certain period of time, but not necessarily for demonstrating efficacy, there may be pressure to conduct studies quickly, and ensuring high quality of research under time pressure can be challenging. Moreover, it was observed that the types of studies conducted under the pediatric exclusivity program tend to match more adult use than actual pediatric needs [37].

Concern was also raised that, as typical for industry-sponsored research, the data belong to the sponsor, and results may not be necessarily or promptly published, or the publications may be influenced by the source of funding [38-41]. Newly enacted legislation tries to address this concern by mandating that the results of all the pediatric studies conducted under the exclusivity program be posted on the FDA Website . More in general, the reported association between sponsorship and research outcome should provide further impetus to supporting publicly funded research programs as a way to both counterbalance and complement industry-funded research.

In addition to research conducted under the pediatric exclusivity program, there has been an increased general interest of pharmaceutical companies in the pediatric market. In fact, the increased pediatric use of medications has created, in some cases, a sizeable enough market to justify funding research programs specifically focused on pediatrics. For instance, the use of medications for ADHD has considerably increased over the years, thus making ADHD the object of novel treatment development, as shown by the introduction into the market of atomoxetine and of several new preparations of stimulants [42]. Likewise, the realization that autism spectrum disorders are much more common than once thought is stimulating industry to conduct research of potential treatments for these conditions.

The Pediatric Research Equity Act of 2003 [9] gives FDA the authority to request that pharmaceutical companies pursuing a new drug registration for adult indications conduct also pediatric studies whenever the medication is potentially relevant to pediatric use. This act is expected to address the need for information in pediatric pharmacology prospectively and proactively, before drugs actually enter the market, thus preventing or minimizing off-label use. The full impact of this initiative will take a few years to be evaluated.

### A Multiple Party Process

Successful implementation of pediatric pharmacology research depends on a close interplay among multiple parties, and most notably NIH, FDA, industry, and academic investigators [43]. As discussed, for drugs that are currently marketed and still covered by patent, pediatric studies can be conducted by industry under agreement with the FDA in return for a six-month extension of patent exclusivity. For drugs currently marketed but already off-patent, the NIH and FDA collaborate towards reviewing the need for pediatric research and preparing an annual priority list, based on which FDA requests specific studies of industry. However, because these drugs are off patent, there are few financial incentives to conduct research, and these requests are often rejected. In these cases, NIH takes responsibility for organizing and funding the studies. This process was recently followed for funding a series of studies that are now in progress to evaluate the pharmacokinetics and efficacy of lithium in the treatment of children with bipolar disorder [44,45].

Finally, for drugs that are still in a pre-registration phase of development and not yet marketed, FDA can request pediatric studies as appropriate. Thus, the recently introduced regulations provide a truly comprehensive process that covers medications in different stages of development and marketing.

While integration and coordination of efforts and activities between government agencies and industry is critical, there are other parties whose role is essential for pediatric pharmacology research. Designing and conducting pediatric research require specific knowledge of methodology and bioethics, and therefore rely on the availability of a properly trained cadre of investigators who have acquired the necessary expertise. Even though the number of pediatric investigators has increased over recent years, pediatric pharmacology remains a relatively small field with limited capacity.

Another critical component is the participation of children and their families in pediatric research. Children are considered a vulnerable population for research purposes and special regulations must be followed for conducting pediatric research [46]. The recent expansion of pediatric pharmacology research has brought attention to the need for better understanding the critical elements of child research participation and improving the implementation and validity of the relevant bioethical procedures [47]. Enrollment in clinical trials is often a slow process that takes several years to be completed. Various strategies for engaging both practitioners and potential research participants have been proposed, and greater attention on the part of researchers to the perspectives and needs of children and families have been recommended [43,48].

## Conclusion

Knowledge on the effects of medications in children has significantly expanded in recent years due to an increase in publicly funded research and legislative initiatives providing financial incentives to industry. Further progress will depend on coordination and integration of efforts across the different parties. It will be especially important to sustain and expand the research infrastructure that has been built over recent years, and develop more rapid and efficient ways of conducting clinical trials in pediatric populations. It will be also critical to increase the collaborations and coordination of efforts between the U.S., Europe, and other countries with the aim of achieving greater standardization of research methodology and sharing of research data, The specific research agenda will depend in large part on the emergence of novel, promising treatments, and on the identification of timely clinical questions of major public health relevance. Clearly, both a public and private vigorous involvement in pediatric research will be needed to address the diverse and complex medical needs of children.

## Competing interests

The author declares that he has no competing interests.
